# Dangers Incurred by the X-Ray Operator

**Published:** 1920-03

**Authors:** Charles A. Eller


### DANGERS INCURRED BY THE X-RAY OPERATOR

This is to be a little heart-to-heart personal talk to all who are interested in the X-ray. I am not telling this story because of any animosity towards any one in particular, but am telling it "so my fellow-dentists may know."

Two years ago last May I purchased an X-ray machine, the best I could buy for dental use. It came, was a beauty and in due time it was installed. I was taught to use it. I loved it. It worked and does yet beautifully. In talking over proper equipment for this machine the question of proper protection was talked about and I was assured over and over again that I would not need a lead screen, other than the lead glass tube bowl or protector that is a part of the equipment.

Thinking, of course, that the men who manufacture and sell the machine ought to know, I did not bother any more about it, and everything went along in smooth order. I was getting good results and was very much pleased.

In May of last year (1918) I noticed a slight breaking out of the fingers of my right hand. This eruption resembled an ivy poison eruption. It was noticeably worse between the fingers. I asked a doctor's opinion about it and he said to paint it with iodine, which I did, but which did no good. It ran on for a couple of weeks and finally became better, and by the 25th of June was quite all gone.

I did not know what had caused the trouble, but was glad to be rid of it as it was quite annoying, though it had not kept me from operating, but hindered considerably. I went along then for a week or so and on Monday, July 9th, the same fingers broke out again with a similar eruption, though noticeably worse. Also thumb and two fingers of left hand. Thumb and all fingers of right hand.

I went on working, but the eruption became so bad by Saturday, the 14th, that I had to close my office. I had an infection from this eruption, accompanied by a temperature of 100 degrees, before I succeeded in getting treatment to stop same. I was away from the office a week when my fingers were nearly well again. I worked for the rest of July and the eruption came again as before and I had to close my office. Each time I would work a day and then in the evening the fingers would blister. I tried three times and each time would have to quit. Finally, after being out of the office eight whole weeks, I tried again, and that night they blistered again. I decided then that it was the X-rays causing the trouble. I have not made an exposure since the first day of October and my hands have been improving constantly since that time.

Conclusion: The X-ray certainly had an accumulative effect.

Use it a little every day and the effect piles up until in my case it took just one year for it to manifest itself in any way.

In January of 1919 my hands were practically well, yet the skin of the fingers of the right hand would crack quite easily and bother me considerably in that way. Just as it takes time for effects of the X-ray to manifest itself, apparently so does it take time for it to leave the body. My left hand, which was not quite so bad, was quite well in January of 1919, while the right hand, which was worse, was still getting better.

I have sold my machine and have my work done away from the office, and though it's harder to do things as I want to, I would rather have my hands than the X-ray machine and no hands.

The manufacturers certainly make a great mistake when they do not insist upon men using proper lead screen protection. It would not cost them more and would mean added patronage in the future. The man that says there is no danger is ignorant of the effects or is trying to sell equipment, not caring for results to his customer.

Of course some people may and must be supersensitive to rays, but it only takes time to find out who they are, and by that time the danger is done at least temporarily.

When I equip again with a machine I shall certainly fix it so that it will be impossible for me to turn on rays except I stand in a lead cabinet that excludes rays from every side and the top. I will take no chances in the future. I would not do even that much under a year or perhaps two. The symptoms were hardly the same as any text-book describes on dental X-rays but nearly exactly describes my case.

One doctor who has charge of a T. B. sanitarium, in talking of symptoms, hit the nail when he said, "Symptoms, hell! The book says nothing about headaches, and yet every time I take a chest picture I get a headache."

I described my case to an eminent X-ray man in Baltimore, Md. This man has lost a finger or two—his hands are excellent samples of what X-ray burns will do for a man—yet he was sure I did not have an X-ray burn. Perhaps I did not have, but I certainly had an acute X-ray dermatitis that if kept up would have resulted in a burn and perhaps cancer of hand or hands. After talking to this man I was in Birmingham, Ala., and visited a dental

X-ray specialist who did not have complete lead protection, and his hands were excellent duplicates of what mine were at their worst stage.

This backing up of my own case is sufficient proof to me, wiser men notwithstanding, that I had at least a case of acute X-ray dermatitis.—Charles A. Eller, in *Dental Health.*

